# Malignancy in systemic lupus erythematosus: relation to disease characteristics in 92 patients – a single center retrospective study

**DOI:** 10.1007/s00296-024-05623-3

**Published:** 2024-06-08

**Authors:** Joanna Kosałka-Węgiel, Renata Pacholczak-Madej, Radosław Dziedzic, Andżelika Siwiec-Koźlik, Magdalena Spałkowska, Mamert Milewski, Lech Zaręba, Stanisława Bazan-Socha, Mariusz Korkosz

**Affiliations:** 1 Department of Rheumatology and Immunology, Jagiellonian University Medical College, Jakubowskiego 2, Kraków, 30-688 Poland; 2 Department of Rheumatology, Immunology and Internal Medicine, University Hospital, Jakubowskiego 2, Kraków, 30-688 Poland; 3 Department of Gynaecological Oncology, Maria Sklodowska-Curie National Research Institute of Oncology, Kraków Branch, Garncarska 11, Kraków, 31-115 Poland; 4 Department of Chemotherapy, The District Hospital, Szpitalna 22, Sucha Beskidzka, 34-200 Poland; 5 Department of Anatomy, Jagiellonian University Medical College, Kopernika 12, Kraków, 31-034 Poland; 6 Doctoral School of Medical and Health Sciences, Jagiellonian University Medical College, Św. Łazarza 16, Kraków, 31-530 Poland; 7 Department of Dermatology, Jagiellonian University Medical College, Botaniczna 3, Kraków, 31-501 Poland; 8 College of Natural Sciences, Institute of Computer Science, University of Rzeszów, Pigonia 1, Rzeszów, 35-310 Poland; 9 Department of Internal Medicine, Faculty of Medicine, Jagiellonian University Medical College, Jakubowskiego 2, Kraków, 30-688 Poland

**Keywords:** Systemic lupus erythematosus, Cancer, Malignancy

## Abstract

**Objective:**

Systemic lupus erythematosus (SLE) is a chronic autoimmune disease with a variable clinical manifestation, potentially leading to death. Importantly, patients with SLE have an increased risk of neoplastic disorders. Thus, this study aimed to comprehensively evaluate the clinical and laboratory characteristics of patients with SLE and with or without malignancy.

**Methods:**

We conducted a retrospective analysis of medical records of 932 adult Caucasian patients with SLE treated at the University Hospital in Kraków, Poland, from 2012 to 2022. We collected demographic, clinical, and laboratory characteristics, but also treatment modalities with disease outcomes.

**Results:**

Among 932 patients with SLE, malignancy was documented in 92 (9.87%), with 7 (7.61%) patients experiencing more than one such complication. Non-hematologic malignancies were more prevalent (*n* = 77, 83.7%) than hematologic malignancies (*n* = 15, 16.3%). Patients with SLE and malignancy had a higher mean age of SLE onset and a longer mean disease duration than patients without malignancy (*p* < 0.001 and *p* = 0.027, respectively). The former group also presented more frequently with weight loss (odds ratio [OR] = 2.62, 95% confidence interval [CI] 1.61–4.23, *p* < 0.001), fatigue/weakness (OR = 2.10, 95% CI 1.22–3.77, *p* = 0.005), and fever (OR = 1.68, 95% CI 1.06–2.69, *p* = 0.024). In the malignancy-associated group, we noticed a higher prevalence of some clinical manifestations, such as pulmonary hypertension (OR = 3.47, 95% CI 1.30–8.42, *p* = 0.007), lung involvement (OR = 2.64, 95% CI 1.35–4.92, *p* = 0.003) with pleural effusion (OR = 2.39, 95% CI 1.43–3.94, *p* < 0.001), and anemia (OR = 2.24, 95% CI 1.29–4.38, *p* = 0.006). Moreover, the patients with SLE and malignancy more frequently had internal comorbidities, including peripheral arterial obliterans disease (OR = 3.89, 95% CI 1.86–7.75, *p* < 0.001), myocardial infarction (OR = 3.08, 95% CI 1.41–6.30, *p* = 0.003), heart failure (OR = 2.94, 95% CI 1.30–6.17, *p* = 0.005), diabetes mellitus (OR = 2.15, 95% CI 1.14–3.91, *p* = 0.011), hypothyroidism (OR = 2.08, 95% CI 1.29–3.34, *p* = 0.002), arterial hypertension (OR = 1.97, 95% CI 1.23–3.23, *p* = 0.003), and hypercholesterolemia (OR = 1.87, 95% CI 1.18-3.00, *p* = 0.006). Patients with SLE and malignancy were treated more often with aggressive immunosuppressive therapies, including cyclophosphamide (OR = 2.07, 95% CI 1.30–3.28, *p* = 0.002), however median cumulative cyclophosphamide dose in malignancy-associated SLE subgroup was 0 g (0–2 g). Interestingly, over a median follow-up period of 14 years (ranges: 8–22 years) a total of 47 patients with SLE died, with 16 cases (5.28%) in the malignancy-associated SLE group and 31 cases (5.73%) in the non-malignancy SLE group (*p* = 0.76). The most common causes of death were infections (21.28%) and SLE exacerbation (8.51%).

**Conclusion:**

The study highlights the relatively frequent presence of malignancies in patients with SLE, a phenomenon that demands oncological vigilance, especially in patients with a severe clinical course and comorbidities, to improve long-term outcomes in these patients.

## Introduction

Systemic lupus erythematosus (SLE) is a chronic autoimmune disease, typified by various clinical manifestations that involve the skin, cardiovascular, mucosal, gastrointestinal, hematological, neurological, musculoskeletal, pulmonary, and renal systems [1]. Most patients with SLE present a relapsing-remitting course with a higher risk of developing life-threatening complications; these patients require high-intensity immunosuppressive therapy [2]. In general, patients with SLE have an increased risk of all hematologic cancers, such as lymphoma, leukemia, and multiple myeloma, but also other types of solid malignancy, including lung cancer, liver and hepatobiliary cancer, vaginal and vulvar cancer, thyroid cancer, head and neck cancer, renal cancer, and nonmelanoma skin cancer [3, 4]. Interestingly, one of the most common malignancies in patients with SLE is non-Hodgkin lymphoma, with a 3-4-fold increased risk compared with the general population with an aggressive histologic subtype, such as diffuse large B-cell lymphoma [5, 6]. In turn, patients with SLE have a lower risk of breast, ovarian, and endometrial cancer, a phenomenon that might be related to hormonal factors and specific lupus antibodies [7]. For example, anti-DNA antibodies inhibit DNA repair, which might be toxic to cancer cells with DNA repair defects, making the cells more sensitive to DNA-damaging agents such as doxorubicin and radiation [8]. According to the recent meta-analysis by Zhang et al. [9], cancers occur in patients with SLE 62% more often than in the general population. In a recent study on malignancies in SLE by Kariniemi et al. [10], patients with SLE and malignancy had a lower adjusted 15-year survival than controls with malignancy, 27.1% versus 52.4%.

Investigations into the relationship between cancer risk and specific SLE disease characteristics, such as organ involvement, disease activity, and treatment regimens, are essential for a comprehensive understanding of this complex interplay. Clinical and laboratory biomarkers might serve as valuable indicators of cancer risk. Therefore, we aimed to analyze differences in disease characteristics and laboratory findings between patients with SLE and with or without cancer to search for potential predictors and risk profiles in patients with SLE and malignancy. Exploring the similarities and differences in cancer risk profiles between individuals with SLE and the general population can provide insights into the unique factors contributing to oncogenesis in the context of autoimmune diseases.

## Patients and methods

### Study population

We retrospectively reviewed medical records of all SLE cases fulfilling the European League Against Rheumatism and American College of Rheumatology (EULAR/ACR) criteria from 2019 [11], who were treated in the University Hospital, Kraków, Poland, from January 2012 to June 2022. Detailed demographic, clinical, and laboratory characteristics of the enrolled patients with SLE are included in our previous publication [12]. In brief, we collected data on sex, current age, age at the first SLE symptoms and disease onset, time delay between the onset of SLE symptoms and diagnosis, duration of the disease, family history of autoimmune diseases, clinical and laboratory SLE manifestations, cause and age of death (if applicable), internal comorbidities, miscarriages in women, and different immunosuppressive treatment modalities. Next, we divided all patients into two subgroups: The first group comprised patients with SLE and a malignancy diagnosis (the malignancy-associated SLE group), whereas the second group consisted of patients with SLE but without a malignancy diagnosis (the non-malignancy-associated SLE group). The evaluated clinical manifestations included: general symptoms, lymphadenopathy, skin lesions, oral or nasopharyngeal ulcerations, photosensitivity, joint involvement, serositis, the hematologic domain (leukopenia, lymphopenia, anemia, hemolytic anemia, thrombocytopenia, macrophage activation syndrome [MAS], and thrombotic thrombocytopenic purpura), lupus nephritis (LN), nervous system involvement, Raynaud’s phenomenon, respiratory system involvement, and lupoid hepatitis; they are described in detail in our previous publication [12]. We extended the evaluation of malignancy in patients with SLE to age at the malignancy diagnosis, the duration between malignancy and the SLE diagnosis, and the type of malignancy. We also analyzed medical comorbidities, such as arterial hypertension, diabetes mellitus, hypercholesterolemia, atrial fibrillation, lower extremity peripheral arterial obliterans disease, heart failure, and any thromboembolic events. The treatment modalities included corticosteroids, hydroxychloroquine or chloroquine, azathioprine, methotrexate, cyclosporine, mycophenolate mofetil, cyclophosphamide, sulfasalazine, immunoglobulins intravenously in suppressive doses, and biological agents (belimumab, rituximab, and anifrolumab), currently or in the past. We also checked whether patients had splenectomy or plasmapheresis.

The Bioethics Committee of the Jagiellonian University Medical College approved the research (No: N41/DBS/000936). All procedures adhered to the ethical principles outlined in the Declaration of Helsinki.

### Laboratory analysis

A complete blood cell count, creatinine with estimated glomerular filtration rate (eGFR) determined by the Modification of Diet in Renal Disease formula, 24-hour urine protein excretion, urinary sediment analysis, lipid profile, haptoglobin, direct antiglobulin test, and blood group designation were analyzed using standard laboratory techniques [13]. Anti-nuclear antibodies (ANA) were assessed through indirect immunofluorescence (IIF). Specific antibodies, including anti-SSA, anti-SSB, anti-histone, anti-nucleosome, anti-Smith (Sm), and anti-ribonucleoprotein (RNP), were identified by using enzyme-linked immunosorbent assays (ELISAs) or line-blot immunoassays. Anti-double-stranded DNA (anti-dsDNA) antibodies were assayed via IIF with *Crithidia luciliae* as the substrate. The anti-myeloperoxidase (MPO) and anti-proteinase 3 (PR3) antibodies were evaluated with a standardized ELISA technique. Serum complement levels (C3c and C4) and rheumatoid factor (RF) were determined using nephelometry. In a retrospective analysis, laboratory tests for hypercoagulability were conducted, encompassing assessments for lupus anticoagulant (LA), anti-cardiolipin (aCL), and anti-beta-2-glycoprotein I (aβ2GPI) antibodies in both the IgM and IgG classes. In addition, antithrombin activity, protein C activity, free protein S level, and factor VIII activity were evaluated, and factor V Leiden and prothrombin G20210A gene variants were identified. All measurements were performed using standard routine laboratory techniques.

### Statistical analysis

We analyzed the results by using STATISTICA Tibco 13.3 and the R software. Categorical variables are presented as frequencies (number of cases) with relative frequencies (percentages), and were compared using the Chi^2^ test or Fisher’s exact test. The normality of the data distribution was evaluated using the Shapiro-Wilk test. All continuous variables were non-normally distributed; thus, they are presented as the median with Q1-Q3 ranges and were compared using the Mann-Whitney test. We calculated the odds ratio (OR) with 95% confidence interval (CI) by determining the cut-off points based on receiver operating characteristic (ROC) curves. We considered a two-sided *p* < 0.05 to be statistically significant for all analyses.

## Results

### Demographic features

The study included 92 patients with SLE and malignancy, constituting 9.87% of the total SLE cases (*n* = 932). Additionally, 7 (7.61%) of the patients with SLE and malignancy had at least two malignancies in their medical records.

Table 1 presents the cumulative demographic characteristics of the SLE cohort. The sex distribution between the malignancy-associated and non-malignancy-associated SLE groups was not significantly different (*p* = 0.72). Patients with confirmed malignancy were diagnosed with SLE a median of 13 years earlier than those without malignancy (46 and 33 years, respectively, *p* < 0.001), but the disease duration was comparable between the analyzed groups (14 and 12 years, respectively, *p* = 0.40). The time delay between symptom onset and diagnosis was similar in both SLE groups (median of 0 months for both, *p* = 0.09), while the first symptoms in the malignancy-associated group appeared at a median of 12 years earlier than in the non-malignancy-associated group (42 and 30 years, respectively, *p* < 0.001).

**Table 1:** Demographic characteristics of the patients with systemic lupus erythematosus and with or without malignancy

Characteristics	Malignancy-associated SLE (*n* = 92)	Non-malignancy-associated SLE (*n* = 840)	*p*-value
Age of onset			
Adult onset (age of onset ≥ 18 years), *n* (%)	87 (94.56%)	749 (90.56%)	0.21
Juvenile onset (age of onset < 18 years), *n* (%)	5 (6.54%)	78 (9.43%)	
General characteristics			
Sex, female, *n* (%)	80 (86.96%)	747 (88.82%)	0.72
Age at SLE onset			
Median (Q1-Q3), years	46 (31-58.5)	33 (24–46)	< 0.001*
Age at first SLE symptoms			
Median (Q1-Q3), years	42 (27-55.5)	30 (22–43)	< 0.001*
Time delay between onset of symptoms and SLE diagnosis			
Median (Q1-Q3), years	0 (0–1)	0 (0–2)	0.09
Age at last visit			
Median (Q1-Q3), years	62 (54-68.5)	48 (37–60)	< 0.001*
SLE duration			
Median (Q1-Q3), years	14 (6–27)	12 (6–19)	0.40

The categorical variables are presented as numbers with percentages, and continuous variables are presented as the median with Q1-Q3 ranges. An asterisk indicates a statistically significant difference

Among the patients with SLE, 157 (17.05%) had a positive family history of any autoimmune diseases. In the malignancy-associated and non-malignancy-associated SLE groups, rheumatoid arthritis, SLE, psoriasis, and Hashimoto’s disease were the most prevalent, with no significant differences between the groups (*p* > 0.05 for all). Other autoimmune diseases included systemic sclerosis, granulomatosis with polyangiitis, mixed connective tissue disease, Sjögren’s syndrome, ulcerative colitis, celiac disease, Graves’ disease, myasthenia gravis, immune thrombocytopenia, autoimmune hepatitis, dermatomyositis, Addison-Biermer anemia, and type I diabetes mellitus; they also showed no significant differences between the groups (*p* > 0.05 for all).

### A more severe course of SLE in patients with an accompanying malignancy

Table 2 provides an overview of the cumulative frequencies of systemic involvement in the SLE cohort. In the malignancy-associated SLE group, the most prevalent clinical manifestations included hematological symptoms (93.42%), joint issues (90.22%), and general symptoms (89.13%). The non-malignancy-associated SLE group exhibited comparable predominant symptoms, with hematological symptoms (91.2%), joint problems (89.89%), and mucocutaneous manifestations (83.12%) being the most frequent. A comparison between the malignancy-associated and non-malignancy-associated SLE groups revealed that the former often presented with more severe clinical manifestations. Specifically, patients with SLE and malignancy exhibited a higher incidence of weight loss (OR = 2.62, 95% CI 1.61–4.23, *p* < 0.001), pleural effusion (OR = 2.39, 95% CI 1.43–3.94, *p* < 0.001), fever (OR = 1.68, 95% CI 1.06–2.69, *p* = 0.024), fatigue/weakness (OR = 2.62, 95% CI 1.61–4.23, *p* < 0.001), and anemia (OR = 2.24, 95% CI 1.29–4.38, *p* = 0.006) compared with patients with SLE but not malignancy. Moreover, pulmonary hypertension and lung involvement were more prevalent in patients with SLE and malignancy (OR = 3.47, 95% CI 1.30–8.42, *p* = 0.007, and OR = 2.64, 95% CI 1.35–4.92, *p* = 0.003, respectively). Interestingly, there were no differences in mucocutaneous symptoms, joint involvement, pericardial effusion, pericarditis, hematological signs (except for anemia), blood ABO groups, Rh blood types (data not shown), kidney involvement, neurological abnormalities, Raynaud’s phenomenon, and lupoid hepatitis (*p* > 0.05 for all).

**Table 2:** The cumulative frequencies of systemic involvement in patients with systemic lupus erythematosus with or without malignancy (symptoms presented at any time in medical history)

Clinical manifestations	Malignancy-associated SLE (*n* = 92)	Non-malignancy-associated SLE (*n* = 840)	*p*-value
Constitutional manifestations, *n* (%)	82 (89.13%)	654 (77.76%)	0.016*
Fever, *n* (%)	50 (56.18%)	353 (43.21%)	0.013*
Fatigue/weakness, *n* (%)	71 (78.89%)	524 (63.98%)	0.007*
Myalgias, *n* (%)	41 (45.56%)	307 (37.58%)	0.17
Weight loss, *n* (%)	36 (40.45%)	168 (20.56%)	< 0.001*
Lymphadenopathy, *n* (%)	24 (26.97%)	154 (18.78%)	0.09
Mucocutaneus manifestations, *n* (%)	73 (79.35%)	699 (83.12%)	0.44
Lupus malar rash, *n* (%)	38 (41.30%)	381 (45.68%)	0.49
Discoid rash, *n* (%)	9 (9.78%)	65 (7.80%)	0.64
Urticaria, *n* (%)	7 (7.61%)	70 (8.4%)	0.95
Cutaneous vasculitis, *n* (%)	8 (8.7%)	53 (6.36%)	0.53
Alopecia, *n* (%)	20 (21.74%)	229 (27.46%)	0.29
Oral and/or nasal ulcers, *n* (%)	20 (21.74%)	124 (14.89%)	0.17
Photosensitivity, *n* (%)	31 (33.7%)	291 (34.93%)	0.90
Other skin changes, *n* (%)	61 (66.30%)	573 (68.46%)	0.76
Joint manifestations, *n* (%)	83 (90.22%)	756 (89.89%)	0.93
Arthritis, *n* (%)	56 (62.22%)	532 (63.79%)	0.86
Arthralgia, *n* (%)	82 (89.13%)	752 (89.74%)	0.99
Serositis, *n* (%)	37 (40.20%)	201 (23.9%)	< 0.001*
Pleural effusion, *n* (%)	29 (31.87%)	137 (16.33%)	< 0.001*
Pericardial effusion, *n* (%)	18 (19.78%)	119 (14.39%)	0.22
Pericarditis, *n* (%)	5 (5.49%)	33 (3.93%)	0.66
Hematological manifestations, *n* (%)	86 (93.48%)	767 (91.20%)	0.59
Leucopenia^1^, *n* (%)	62 (68.89%)	515 (62.65%)	0.29
Lymphopenia^2^, *n* (%)	53 (58.89%)	420 (52.57%)	0.46
Anemia^3^, *n* (%)	76 (84.4%)	583 (70.75%)	0.009*
Hemolytic anemia^4^*n* (%)	10 (27.78%)	77 (20.59%)	0.43
Thrombocytopenia^5^, *n* (%)	34 (37.36%)	262 (31.87%)	0.34
Direct Coombs test, *n* (%)	5 (23.81%)	57 (41.61%)	0.19
Macrophage activation syndrome, *n* (%)	2 (2.2%)	6 (0.72%)	0.40
Thrombotic thrombocytopenic purpura, *n* (%)	1 (1.09%)	1 (0.12%)	0.47
Kidney involvement, *n* (%)	33 (35.87%)	345 (41.02%)	0.40
24-hour urinary protein excretion > 0.5 g/day, *n* (%)	30 (34.09%)	290 (35.54%)	0.88
24-hour urinary protein excretion > 3.5 g/day, *n* (%)	16 (19.05%)	147 (18.94%)	0.90
Urinary casts, *n* (%)	17 (20.48%)	124 (16.96%)	0.52
Erythrocyturia, *n* (%)	25 (29.41%)	224 (28.79%)	0.99
Leukocyturia, *n* (%)	29 (33.72%)	253 (31.90%)	0.82
Neurological abnormality, *n* (%)	9 (9.78%)	95 (11.30%)	0.79
Central nervous system involvement, *n* (%)	3 (3.26%)	67 (8.00%)	0.15
Peripheral nervous system involvement, *n* (%)	7 (7.61%)	40 (4.78%)	0.36
Raynaud’s phenomenon, *n* (%)	20 (21.74%)	212 (25.30%)	0.53
Lung involvement, *n* (%)	16 (17.39%)	62 (7.37%)	0.002*
Interstitial lung disease, *n* (%)	9 (9.78%)	39 (4.65%)	0.06
Diffuse alveolar hemorrhage, *n* (%)	2 (2.17%)	9 (1.07%)	0.67
Pulmonary hypertension, *n* (%)	8 (8.70%)	22 (2.66%)	0.005*
Lupoid hepatitis, *n* (%)	5 (5.43%)	40 (4.77%)	0.98

The categorical variables are presented as numbers with percentages. An asterisk indicates a statistically significant difference. ^1^ < 4000/mm^3^ or diagnosis based on medical history; ^2^ < 1500/mm^3^ or diagnosis based on medical history; ^3^ ≤ 12 g/dL in women, ≤ 13.5 g/dL in men, or diagnosis based on medical history; ^4^ anemia with a positive direct Coombs test or anemia with a decreased level of haptoglobin or diagnosis based on medical history; ^5^ <100,000/mm^3^ or diagnosis based on medical history

There were no differences in the autoantibody profile (Table 3), except for anticardiolipin antibodies in the IgG class, which were documented more frequently as positive in the non-malignancy-associated SLE group (*p* = 0.002). For example, anti-PR3 antibodies were examined in 144 (15.45%) out of 932 SLE patients, and anti-MPO antibodies were assessed in 151 (16.2%) out of 932 SLE patients. Anti-PR3 antibodies tested positive in only 7 (4.86%) of all SLE patients, whereas anti-MPO antibodies were positive in 14 (9.27% of all SLE patients) without statistically significant differences between SLE groups (*p* > 0.05 for both). Moreover, the malignancy-associated and non-malignancy-associated SLE groups did not differ in antithrombin activity, protein C activity, the free protein S level, factor VIII activity, factor V Leiden gene mutations, and G20210A prothrombin gene mutations (data not shown).

**Table 3:** The laboratory findings in patients with systemic lupus erythematosus and with or without malignancy

Laboratory parameter (number of patients with the analyzed parameter)	Malignancy-associated SLE (*n* = 92)	Non-malignancy-associated SLE (*n* = 840)	*p*-value
Rheumatoid factor, *n* (%)	20 (32.79%)	145 (32.58%)	0.88
ANA – IIF assay, *n* (%)	92 (100%)	840 (100%)	0.99
Anti-SSA antibodies^1^, *n* (%)	50 (58.14%)	474 (59.92%)	0.84
Anti-SSB antibodies^1^, *n* (%)	26 (30.23%)	228 (28.82%)	0.88
Anti-histone antibodies^1^, *n* (%)	21 (24.42%)	223 (28.19%)	0.54
Anti-nucleosome antibodies^1^, *n* (%)	27 (31.40%)	278 (35.15%)	0.57
Anti-Smith antibodies^1^, *n* (%)	8 (9.30%)	104 (13.20%)	0.39
Anti-RNP antibodies^1^, *n* (%)	14 (16.28%)	179 (22.69%)	0.22
Anti-dsDNA antibodies^1^, *n* (%)	27 (31.76%)	312 (39.64%)	0.19
Anti-dsDNA antibodies^2^, *n* (%)	52 (61.18%)	544 (71.77%)	0.06
Anti-PR3 antibodies^3^, *n* (%)	5 (6.49%)	2 (2.99%)	0.34
Anti-MPO antibodies^3^, *n* (%)	9 (11.11%)	5 (7.14%)	0.38
Antiphospholipid antibodies			
Lupus anticoagulant, *n* (%)	12 (18.46%)	185 (30.43%)	0.06
Anti-cardiolipin antibodies IgG and/or IgM, *n* (%)	29 (41.43%)	398 (58.62%)	0.008*
Anti-cardiolipin antibodies IgG, *n* (%)	17 (24.64%)	296 (44.11%)	0.002*
Anti-cardiolipin antibodies IgM, *n* (%)	21 (30.00%)	273 (40.93%)	0.08
Anti-β2 glycoprotein I IgG and/or IgM, *n* (%)	14 (23.73%)	144 (26.62%)	0.76
Anti-β2 glycoprotein I IgG, *n* (%)	5 (8.62%)	92 (27.49%)	0.09
Anti-β2 glycoprotein I IgM, *n* (%)	13 (21.67%)	95 (18.20%)	0.45
Lupus anticoagulant, or anti-cardiolipin antibodies IgG and/or IgM, or anti-β2 glycoprotein I IgG and/or IgM, *n* (%)	35 (57.38%)	429 (70.91%)	0.04*
Lupus anticoagulant, and anti-cardiolipin antibodies IgG and/or IgM, and anti-β2 glycoprotein I IgG and/or IgM, n(%)	4 (4.35%)	97 (11.53%)	0.05

The categorical variables are presented as numbers with percentages. An asterisk indicates a statistically significant difference. ^1^ Immunoblotting assay; ^2^ the *Crithidia luciliae* immunofluorescence test (CLIFT); ^3^ enzyme-linked immunosorbent assay. Abbreviations: ANA – antinuclear antibodies; dsDNA – double-stranded DNA; IIF – indirect immunofluorescence; MPO – myeloperoxidase; PR3 – proteinase 3; RNP – ribonucleoprotein

Throughout the median follow-up period of 14 years (ranges: 8–22 years), a total of 47 patients with SLE died, with 16 cases (5.28%) in the malignancy-associated SLE group and 31 cases (5.73%) in the non-malignancy SLE group (*p* = 0.76). Among the deceased, the most common causes of death were infections (6 cases, 37.5% vs. 4 cases, 12.9%) and SLE exacerbations (3 cases, 18.75% vs. 1 case, 3.23%), with no difference between the malignancy-associated and non-malignancy-associated SLE subgroups.

### A higher frequency of internal comorbidities in patients with SLE and malignancy

The frequency of internal comorbidities was higher in the malignancy-associated SLE group compared with the non-malignancy-associated SLE group (Table 4). Specifically, patients with SLE and malignancy exhibited a higher occurrence of peripheral arterial obliterans disease (OR = 3.89, 95% CI 1.86–7.75, *p* < 0.001), heart failure (OR = 2.94, 95% CI 1.30–6.17, *p* = 0.005), diabetes mellitus (OR = 2.15, 95% CI 1.14–3.91, *p* = 0.011), hypothyroidism (OR = 2.08, 95% CI 1.29–3.34, *p* = 0.002), arterial hypertension (OR = 1.97, 95% CI 1.23–3.23, *p* = 0.003), and hypercholesterolemia (OR = 1.87, 95% CI 1.18-3.00, *p* = 0.006). Furthermore, myocardial infarction was diagnosed more frequently in the malignancy-associated SLE group compared with the non-malignancy-associated SLE group (OR = 3.08, 95% CI 1.41–6.30, *p* = 0.003). There were no significant differences in other arterial and venous thrombotic episodes between the groups.

**Table 4:** The cumulative frequencies of comorbidities in patients with systemic lupus erythematosus and with or without malignancy

Comorbidities^1^	Malignancy-associated SLE (*n* = 92)	Non-malignancy-associated SLE (*n* = 840)	*p*-value
Arterial hypertension, *n* (%)	62 (67.39%)	430 (51.13%)	0.004*
Diabetes mellitus, *n* (%)	17 (18.48%)	80 (9.51%)	0.012*
Heart failure^2^, *n* (%)	11 (11.96%)	37 (4.40%)	0.004*
Hypercholesterolemia^3^, *n* (%)	57 (61.96%)	391 (46.55%)	0.007*
Atrial fibrillation, *n* (%)	5 (5.43%)	28 (3.33%)	0.46
Peripheral artery disease, *n* (%)	14 (15.22%)	37 (4.40%)	< 0.001*
End-stage renal disease, *n* (%)	3 (3.26%)	23 (2.74%)	0.96
Monoclonal gammopathy of undetermined significance, *n* (%)	19 (20.65%)	0 (0.0%)	< 0.001*
Artery thrombotic episode, *n* (%)	7 (7.61%)	92 (10.94%)	0.33
Stroke, *n* (%)	5 (5.43%)	70 (8.32%)	0.44
Transient ischemic attack, *n* (%)	0 (0.0%)	16 (1.90%)	0.36
Myocardial infarction, *n* (%)	12 (13.04%)	39 (4.64%)	0.002*
Thrombotic episode in another artery, *n* (%)	3 (3.26%)	19 (2.26%)	0.81
Venous thrombotic episode, *n* (%)	23 (25%)	150 (17.84%)	0.09
Deep venous thrombosis, *n* (%)	19 (20.65%)	124 (14.76%)	0.18
Pulmonary embolism, *n* (%)	5 (5.43%)	17 (2.02%)	0.29
Deep venous thrombosis and pulmonary embolism, *n* (%)	4 (4.35%)	17 (2.02%)	0.29
Thrombotic episode in another venous, *n* (%)	4 (4.35%)	15 (1.78%)	0.21
Miscarriage, *n* (%)^4^	11 (16.92%)	101 (16.21%)	0.88

The categorical variables are presented as numbers with percentages. An asterisk indicates a statistically significant difference. ^1^ Current or past comorbidities; ^2^ symptoms of heart failure or left ventricular ejection fraction ≤ 40% or diagnosis based on medical history; ^3^ low-density lipoprotein > 3 mmol/L or pharmacotherapy with statin or diagnosis based on medical history; ^4^% of women with miscarriage from number of women with SLE

### Patients with SLE and malignancy were treated more aggressively before their malignancy diagnosis

Next, we delved into the various approaches to immunosuppressive therapy (Table 5). Steroids were the most commonly administered treatment for both the malignancy-associated and non-malignancy-associated SLE groups (94.51% and 96.18%, respectively, *p* = 0.62). The malignancy-associated SLE group was more frequently prescribed a more aggressive immunosuppressive regimen involving cyclophosphamide, which was utilized more often in this group (OR = 2.07, 95% CI 1.30–3.28, *p* = 0.002). In malignancy-associated SLE patients, the median cumulative dose of cyclophosphamide was 0 g (range: 0–2 g). Conversely, hydroxychloroquine was administered less frequently in the malignancy-associated SLE group compared with the non-malignancy-associated SLE group (OR = 0.62, 95% CI 0.39–0.98, *p* = 0.035).

**Table 5:** The treatments received by patients with systemic lupus erythematosus and with or without malignancy

Treatment	Malignancy-associated SLE (*n* = 92)	Non-malignancy-associated SLE (*n* = 840)	*p*-value
Corticosteroids (oral and/or intravenous), *n* (%)	86 (94.51%)	805 (96.18%)	0.62
Chloroquine or hydroxychloroquine, *n* (%)	60 (65.22%)	653 (77.74%)	0.007*
Azathioprine, *n* (%)	35 (38.89%)	333 (40.02%)	0.92
Methotrexate, *n* (%)	16 (17.78%)	174 (20.96%)	0.57
Cyclosporine, *n* (%)	8 (8.89%)	69 (8.31%)	0.99
Belimumab, *n* (%)	1 (1.11%)	40 (4.84%)	0.17
Mycophenolate mofetil, *n* (%)	25 (27.78%)	276 (33.29%)	0.35
Cyclophosphamide, *n* (%)	42 (46.15%)	243 (29.28%)	0.001*
Rituximab, *n* (%)	6 (6.67%)	24 (2.90%)	0.11
Immunoglobulins, *n* (%)	4 (4.44%)	25 (3.02%)	0.67
Plasmapheresis, *n* (%)	5 (5.56%)	26 (3.14%)	0.37
Sulfasalazine, *n* (%)	4 (4.44%)	43 (5.19%)	0.96
Anifrolumab, *n* (%)	0 (0.0%)	10 (1.21%)	0.61
Splenectomy, *n* (%)	0 (0.0%)	5 (0.6%)	0.99

The categorical variables are presented as numbers with percentages. An asterisk indicates a statistically significant difference

### Characteristics of malignancy in patients with SLE

The median age of patients at the time of the malignancy diagnosis was 56 years (48–63 years), with 18 malignancies diagnosed before SLE onset (median patient age 47.5 years [30–56] years), 9 at the same time of SLE diagnosis (median patient age 60 [54–63] years), and 62 reported after SLE diagnosis (median patient age 57.5 [50–65] years). The overall median duration between SLE onset and malignancy diagnosis was 9 (0–20) years. Notably, the distribution of non-hematologic and hematologic malignancies was comparable among the three abovementioned subgroups. Non-hematologic malignancies were more frequent than hematologic malignancies (*n* = 77, 83.7% vs. *n* = 15, 16.3%, *p* < 0.001). Detailed information is provided in Table 6. Among the non-hematologic malignancies, the most prevalent were breast cancer (*n* = 10), thyroid cancer (*n* = 8), and uterine cancer (*n* = 8). For the hematologic malignancies, the most common was non-Hodgkin lymphoma (*n* = 9).

**Table 6:** The types of malignancies observed in 92 patients with systemic lupus erythematosus and malignancy

Malignancy	Number (%)
Hematologic malignancies	
Non-Hodgkin lymphoma, *n* (%)	9 (9.78%)
Hodgkin lymphoma, *n* (%)	2 (2.17%)
Leukemia, *n* (%)	1 (1.09%)
Multiple myeloma, *n* (%)	1 (1.09%)
Essential thrombocythemia, *n* (%)	1 (1.09%)
Polycythemia vera, *n* (%)	1 (1.09%)
All hematologic malignancies, *n* (%)	15 (16.30%)
Non-hematologic malignancies	
Breast cancer, *n* (%)	10 (10.87%)
Thyroid cancer, *n* (%)	8 (8.70%)
Uterine cancer, *n* (%)	8 (8.70%)
Colorectal cancer, *n* (%)	6 (6.52%)
Skin cancer – basal cell carcinoma, *n* (%)	6 (6.52%)
Pancreatic cancer, *n* (%)	5 (5.43%)
Bladder cancer, *n* (%)	4 (4.35%)
Melanoma, *n* (%)	4 (4.35%)
Glioblastoma multiforme, *n* (%)	4 (4.35%)
Hepatobiliary cancer, *n* (%)	2 (2.17%)
Lung cancer, *n* (%)	2 (2.17%)
Thymoma, *n* (%)	2 (2.17%)
Others cancer, *n* (%)	10 (10.87%)
All non-hematologic malignancies, *n* (%)	77 (83.70%)

The categorical variables are presented as numbers with percentages. Other malignancies included: parathyroid gland cancer (*n* = 1), adrenal cortex cancer (*n* = 1), salivary gland cancer (*n* = 1), pleural mesothelioma cancer (*n* = 1), ovarian cancer (*n* = 1), renal cancer (*n* = 1), vagina cancer (*n* = 1), prostate cancer (*n* = 1), vulvar cancer (*n* = 1), and bronchus cancer (*n* = 1)

Compared with patients with non-hematological malignancies, patients with hematological malignancies presented higher rates of LN diagnosed at the onset of SLE or during the first year of disease duration (36.67% vs. 15%, *p* = 0.02). Additionally, patients with hematological malignancies were diagnosed more frequently with monoclonal gammopathy of undetermined significance (MGUS) (60% vs. 1.61%, *p* < 0.001) and MAS (6.67% vs. 0%, *p* = 0.041). The treatment patterns also differed: Patients with hematological malignancies were treated more frequently with immunosuppressive drugs, including cyclosporine (20% vs. 3.33%, *p* = 0.009), mycophenolate mofetil (43.33% vs. 20%, *p* = 0.02), rituximab (16.67% vs. 1.67%, *p* = 0.007), and plasmapheresis (13.33% vs. 1.67%, *p* = 0.023). Furthermore, patients with hematological malignancies exhibited specific manifestations of SLE, including pericardial effusion (33.33% vs. 13.12%, *p* = 0.023), interstitial lung disease (20% vs. 4.84%, *p* = 0.022), and diffuse alveolar hemorrhage (6.67% vs. 0%, *p* = 0.04), more often that patients with non-hematological malignancies.

Patients with non-hematological malignancies manifested distinct features. This group was more often associated with SLE presenting with alopecia (29.03% vs. 6.67%, *p* = 0.015). Interestingly, skin vasculitis was observed only in patients with non-hematological malignancies (12.9%). Additionally, patients with non-hematological malignancies were treated more frequently with chloroquine or hydroxychloroquine (51.67% vs. 23.33%, *p* = 0.01).

Despite these differences, both groups showed similarities in the sex distribution, family history of autoimmune diseases, the mortality index, renal involvement in the course of SLE, the types of ANA and antineutrophil cytoplasmic antibodies (ANCA), and comorbidities, including thromboembolic episodes.

Analysis of the timing of malignancy diagnoses in relation to the timing of the SLE diagnosis revealed disparities between the groups. First, there were cases where malignancies were diagnosed prior to or concurrent with SLE. Second, there were instances where malignancies were diagnosed after SLE. In the first group, the median age at the onset of the first SLE symptoms was higher (54 vs. 37.5 years, *p* < 0.001), and the age at SLE onset was higher (59 vs. 39 years, *p* < 0.001). These subgroups did not differ in terms of LN frequency, but they did differ in the number of LN flares. For example, in the first group LN was diagnosed in 7 cases (25.93%) compared with 25 cases (40.32%) in the second group (*p* = 0.193). In turn, there were more LN flares in the second group (*p* = 0.018). However, the age of LN onset was higher in the first group compared to second one group (63 vs. 44.5 years, *p* < 0.001). In addition, the following were more common in the second group: myocardial infarction (0% vs. 17.74%, *p* = 0.019), treatment with azathioprine (7.41% vs. 53.33%, *p* < 0.001), malar rash (22.22% vs. 50%, *p* = 0.015), joint pain (74.07% vs. 95.16%, *p* = 0.004), and Raynaud’s phenomenon (7.41% vs. 27.42%, *p* = 0.034).

## Discussion

We have provided comprehensive insights into the intricate relationship between SLE and malignancy, shedding light on various aspects, including demographic features, clinical manifestations, comorbidities, laboratory findings, and long-term outcomes. The observed prevalence of malignancies in the SLE cohort underscores the importance of understanding the intersection between these two complex conditions, because different types of malignancies occurred in almost 1 out of 10 patients from our SLE cohort.

Out of 932 patients with SLE, 9.87% had malignancy, with 7.61% of them had multiple malignancies. Patients with SLE and malignancy exhibited an older age at SLE onset and initial symptoms, with a significantly higher median age compared with patients with SLE but not malignancy. This observation is in line with previous reports [14, 15]. Moreover, both subgroups showed no differences in the sex distribution and time delay between symptom onset and diagnosis, with a similar disease duration between the groups. Approximately 70% of all malignancies were detected after a SLE diagnosis, consistent with the current knowledge [16]. However, it is worth highlighting that the risk of malignancy development in SLE is believed to be the highest during early SLE, especially in patients with a disease duration of < 1 year [16].

Interestingly, the pathogenesis of cancer development in patients with SLE is not fully understood [17]. Many studies have investigated the link between malignancy and SLE, pointing out the potential role of genetics and clinical or environmental factors in its development [18]. It has been postulated that an aberrant immune response in SLE not only contributes to the disease manifestations, but may also create an environment conducive for the development of malignancies. Moreover, changes in epigenetic regulations described in SLE might be linked with cancers, particularly regarding DNA methylation, histone modification, and non-coding messenger RNA (mRNA) activation. Furthermore, other mechanisms have been suggested, including high expression of a proliferation-inducing ligand (APRIL), a cytokine from the tumor necrosis factor ligand superfamily. APRIL might enable B cells in non-Hodgkin lymphoma to escape from apoptosis, favoring cancer development [19]. Furthermore, an increased level of interleukin(IL)-6 and IL-10 and the use of immunosuppressive therapy might also be related to the malignancy pathogenesis in SLE [20].

Several studies have consistently reported an increased risk of malignancy among patients with SLE. In a large cohort comprising 30,478 individuals with SLE, 1273 (4.18% of SLE cases) were diagnosed with malignancy. The prevalent malignancy types included breast cancer, lung cancer, colon/rectum cancer, and non-Hodgkin lymphoma. Notably, this cohort also exhibited occurrences of less common cancers such as valvular/vaginal cancer, which are infrequently observed in the general population [21]. However, our SLE cohort showed a higher incidence of malignancy, with almost 10% of patients being diagnosed, a higher rate compared with the study by Parikh-Patel et al. [21]. Furthermore, 7.61% of the enrolled patients with SLE and malignancy experienced at least two distinct malignancies. In a Korean population study [14], the estimated malignancy risk in patients with SLE was 1.45 (95% CI 0.74–2.16), with cervical cancer, non-Hodgkin lymphoma, and bladder cancer being the most frequently observed types. Cader et al. [22] evaluated 228 patients with SLE. Eight individuals were diagnosed with malignancy in its early stages, and they underwent radical treatment without signs of recurrence during the observation period.

Regarding hematological malignancies, our results align with the published data, with non-Hodgkin lymphoma being the most prevalent malignant disease in SLE [14, 21, 22]. In contrast, for non-hematological malignancies, besides breast, colorectal, uterine, and bladder cancers being the most frequently diagnosed; other frequently confirmed malignancies included thyroid, skin, and pancreatic cancers. Intriguingly, unlike the findings reported by Parikh-Patel et al. [21], lung cancer was quite rare in our SLE cohort: It was only diagnosed in two patients with SLE.

We did not observe any differences in a family history of various autoimmune diseases between the malignancy-associated and non-malignancy-associated SLE groups. These findings are consistent with the results reported by Bernatsky et al. [23]. While single case reports suggest a potential influence of a family history of SLE on malignancies in patients with SLE [24], our results are not consistent with these reports. Detailed information on familial factors in such a large SLE cohort, considering autoimmune disorders, is nearly absent in the current literature.

Constitutional manifestations, such as weight loss, fever, and fatigue/weakness, were more frequent in patients with SLE and malignancy. In clinical practice, it is crucial to distinguish these symptoms and to determine whether they are linked to a lupus flare, infection, or malignancy. Additionally, symptoms like anemia or pleural effusion may exist within the spectrum of SLE or indicate the onset of an oncological process. This poses a challenge for clinicians and practitioners [25, 26].

All patients in our SLE cohort exhibited positive ANA identified through IIF. ANA serve as a valuable serological marker for various autoimmune diseases, including SLE. It is important to note that they can also be present in the serum of patients with malignancies [27]. These data suggest that ANA could play a role in the pathogenesis of malignancy, as well as other premalignant diseases [27]. We confirmed the SLE diagnosis based on the EULAR/ACR criteria; therefore, we did not observe differences in the prevalence of ANA (based on IIF) between the SLE subgroups. In the study by Nisihara et al. [28], some patients with malignancy were positive for anti-RNP, anti-Sm, anti-Ro, and anti-La, which is consistent with our results. However, there were no differences in ANA types, as identified by immunoblot assay, between the malignancy-associated and non-malignancy-associated SLE groups. Intriguingly, Ladouceur et al. [19] highlighted the potential role of lupus-related anti-DNA antibodies in reducing the risk of breast cancer by inhibiting DNA repair. Moreover, our findings align with the study by Shah et al. [29], who observed a decreased risk of breast cancer in cases where anti-dsDNA or anti-La antibodies were positive.

Another crucial issue that requires discussion is the association between ANCA and malignancy. We did not observe differences in the frequencies of anti-PR3 and anti-MPO antibodies between the malignancy-associated and non-malignancy-associated SLE groups. Conversely, the current literature indicates that ANCA are produced in malignancy and are not necessarily related to autoimmune vasculitis, as commonly believed [30]. Additionally, ANCA might be associated with a poor prognosis for some cancers, such as Ewing’s sarcoma and cutaneous melanoma [30]. This discrepancy may be linked to the interference of ANA in the differentiation between the perinuclear ANCA (p-ANCA) and cytoplasmic ANCA (c-ANCA) patterns [31], as well as the utilization of ELISAs in a relatively small study group.

Of particular interest concerning antiphospholipid antibodies (aPLA) is the higher incidence of anticardiolipin antibodies of the IgG class in the non-malignancy-associated SLE group. This finding is in contrast to the data presented by Armas et al. [32]. Moreover, the presence of aPLA has been reported in conjunction with various solid and hematologic malignancies [33, 34]. So far, patients with malignancies and aPLA are characterized by a higher incidence of thromboembolic events, but we did not confirm such a relationship in our study [35]. We observed a higher incidence of myocardial infarction in the malignancy-associated SLE group, but without an increased frequency of aPLA in this group. Therefore, myocardial infarction may be a consequence of other factors, including comorbidities associated with SLE.

Despite increased recognition of SLE with more sensitive diagnostic tests, the inclusion of milder cases, earlier diagnosis or treatment, increasingly judicious therapy, and prompt treatment of complications, patients with SLE still have mortality rates that are up to 5.3 times higher than that of the general population [36–41].

Interestingly, the malignancy process did not impact the increasing the mortality risk in our SLE cohort, a finding that is in line with data presented by Yurkovich et al. [42]. These comparable mortality rates with different times of malignancy onset in patients with SLE underscore the persistent need for regular oncological screening in these patients.

Another intriguing finding from our study is the increased incidence of arterial hypertension, diabetes mellitus, heart failure, and hypercholesterolemia in patients with SLE and malignancy. Importantly, SLE and malignancy share common risk factors that accumulate over a lifetime, such as smoking, a sedentary lifestyle, and dietary habits [43]. Metabolic syndrome, which encompasses all these risk factors, is significantly associated with the risk of obesity-related malignancies, particularly pancreatic, postmenopausal breast, endometrial, and colorectal cancers [43, 44]. Inflammation, which is linked to atherosclerosis and diabetes mellitus, also plays a role in carcinogenesis, but this relationship is bidirectional. Conversely, malignancy treatment can induce endothelial damage, further exacerbating atherosclerosis [45]. Endothelial dysfunction has been investigated in other connective tissue diseases and appears to be a hallmark of these conditions [46–49]. Furthermore, molecules that are involved in lipid metabolism, endothelial function, regulation of autophagy, and metastatic process in malignancies have been identified [50–52]. This issue warrants further investigation and underscores the complexity of metabolic pathways and their impact on overall health.

Finally, it is worth discussing the impact of immunosuppressive drugs on oncogenesis in the SLE cohort. Cyclophosphamide is an alkylating agent used as an immunosuppressant and chemotherapeutic. It was administered more often in the malignancy-associated SLE group compared with the non-malignancy-associated SLE group. Despite these properties, it has been found to be carcinogenic, and the risk increases with the length of exposure. The most common malignancies associated with this agent are bladder cancer, skin cancer, and secondary acute leukemia (preceded by myelodysplastic syndrome) [53]. However, researchers have reported conflicting data on this matter in patients with SLE. The findings from a Swedish registry indicated that cases of myeloid leukemia in patients with SLE were not related to cyclophosphamide exposure [54], mirroring the observations reported by Guo et al. [55], who found no discernible associations between drug exposure and malignancy risk. Conversely, Bernatsky et al. [56] suggested an increased risk of hematological malignancies in patients with SLE following exposure to immunosuppressants (hazard ratio [HR] 2.29, 95% CI 1.02–5.15) with cyclophosphamide driving the risk (drug-specific HR for exposure of 3.55, 95% CI 0.94–13.37), although associations with other immunosuppressants were not evident. Another study indicated an increased risk of non-melanoma skin cancer with cyclophosphamide (adjusted HR 15.3, 95% CI 3.03–77.5), but no associations with other cancer types [57]. Notably, the cumulative dose of cyclophosphamide appears to be of particular concern [14].

The protective effect of hydroxychloroquine has been suggested previously, and our study aligns with these findings. Antimalarial drugs have been shown to decrease the risk of breast and non-melanoma skin cancer in patients with SLE [57]. Another study found that hydroxychloroquine was associated with decreased odds of cancer (OR 0.417, 95% CI 0.220–0.791) [55]. Ruis-Irastaroze et al. [58] evaluated the impact of antimalarials on cancer risk in patients with SLE, revealing an 85% reduction in cancer risk among those treated with antimalarials (HR 0.15, 95% CI 0.02–0.99). Hydroxychloroquine has also garnered attention in cancer treatment. Evidence suggests that it may enhance the anti-tumoral response in hormone-positive breast cancer when combined with tamoxifen or faslodex, agents that block estrogen receptors [59]. Furthermore, a phase I trial tested the combination of hydroxychloroquine and erlotinib, an epidermal growth factor receptor (EGFR) tyrosine kinase inhibitor, in EGFR-mutant non-small cell lung cancer; it demonstrated preliminary activity and acceptable tolerability [60]. Additionally, a randomized, double-blind, placebo-controlled trial was performed in patients with glioblastoma multiforme who were treated with standard chemoradiotherapy with chloroquine or placebo after tumor resection. Although the HR did not reach statistical significance (0.52, 95% CI 0.21–1.26, *p* = 0.139), the median survival was 24 months in the chloroquine-treated cohort versus 11 months for the controls [61]. Several potential explanations for these observations have been proposed. One possible anti-tumoral mechanism involved the promotion of autophagy, which plays an important role in cancer cell invasion [62], providing the rationale for combining antimalarials with DNA-damaging chemotherapy [63]. Chloroquine has also been shown to inhibit telomerase, which is involved in the unlimited replication of cancer cells [61], and to increase the synthesis of TP53, a protein whose expression increases in response to genotoxic stimuli or environmental stress [64].

### Study limitations

Our study is subject to several limitations. The retrospective design introduces potential biases related to reliance on historical medical records. The single-center approach raises concerns about external validity, as patient demographics and practices may vary across different settings. The observational nature precludes causal inferences, and unaccounted confounders may influence the outcomes. Retrospective data collection might result in incomplete information, impacting the accuracy of our analysis. Our focus on specific features may have led us to overlook other relevant variables. Addressing these limitations is crucial for interpreting our results and guiding future research in understanding the intricate relationship between SLE and malignancy.

## Conclusions

The comprehensive analysis of demographic features, clinical manifestations, and long-term outcomes in patients with SLE and with or without malignancy provides valuable insights into the multifaceted nature of these conditions in a real-world setting. Our findings underscore the clinical complexity in individuals with SLE who develop malignancy, revealing distinct patterns of comorbidities, immunosuppressive treatment, and clinical manifestations.
